# Formulation and optimization of duloxetine hydrochloride buccal films: *in vitro* and *in vivo* evaluation

**DOI:** 10.1080/10717544.2017.1402216

**Published:** 2017-11-24

**Authors:** Amr Mostafa El Sharawy, Marwa Hassan Shukr, Ahmed Hassen Elshafeey

**Affiliations:** a National Organization for Drug Control and Research (NODCAR), Cairo, Egypt;; b Department of Pharmaceutics and Industrial Pharmacy, Faculty of Pharmacy, Cairo University, Cairo, Egypt

**Keywords:** Buccoadhesive films, duloxetine hydrochloride, mucoadhesion, drug release, accelerated stability, bioavailability

## Abstract

Duloxetine hydrochloride (DH) is a serotonin–norepinephrine reuptake inhibitor (SSNRI) indicated for the treatment of depression. Duloxetine suffers from reduced oral bioavailability (≈50%) due to hepatic metabolism. This study aims to develop DH buccoadhesive films to improve its bioavailability. DH buccoadhesive films were prepared adopting the solvent casting method using hydroxypropyl methylcellulose (HPMC) and polyvinyl alcohol (PVA). The prepared films were evaluated for weight uniformity, drug content, surface pH, swelling index, mucoadhesion strength and drug release percentages. Accelerated stability and bioavailability studies in healthy human volunteers were also performed for the selected films. Results of the evaluation tests showed that the optimum physicochemical characters were obtained by the films prepared with 2% HPMC using 10% propylene glycol (F2 films). Accelerated stability studies revealed that DH showed proved stability throughout the experiment time. DH bioavailability from F2 films was determined and compared with that of the marketed oral capsules (Cymbalta^®^ 30 mg). The pharmacokinetic results showed that *C*_max_ for F2 was higher than the market product. In addition, ANOVA analysis showed that a *T*_max_ of F2 film was significantly lower, while, the AUC_0–72_ of F2 was significantly higher than that of Cymbalta capsules. The percentage relative bioavailability of DH from F2 was found to be 296.39%. Therefore, the prepared buccal films offer an alternative route for the administration of DH with the possibility of improving its bioavailability.

## Introduction

Oral mucosal cavity has been considered as potential site for drug administration. This transmucosal route of drug delivery offers several advantages over oral administration for systemic drug delivery including the bypass of the first pass effect and avoidance of possible degradation in the gastrointestinal tract by the gastric fluid, in addition to high patient compliance (Patel et al., 2011). Buccal drug delivery for the systemic circulationprovides a number of advantages such rapid onset of action, sustained delivery, highpermeability, high blood flow, and is easily accessible for both application and removal of a drug delivery device (Madhav et al., 2009). Various mucoadhesive mucosal dosage forms have been developed, which included adhesive films (Singh et al., 2008).

Mucoadhesive films have gained importance due to their acceptable pharmaceutical and medicinal properties (Morales & McConville, 2011; Bala et al., 2013). Mucoadhesive buccal films have the advantages of having improved patient compliance because of their small size and thickness as compared to conventional tablets (Dixit & Puthli, 2009). Moreover, the dose can be taken at the specified time at any place. The films can be designed to dissolve upon contact with a wet surface, such as tongue, within few seconds (fast-dissolving films) or can be designed to release the drug over a specific period of time depending upon the formulation design. Buccoadhesive films exhibit the advantage of avoiding the first pass effect by directing absorption through the venous system that drains from the cheek (7).

Duloxetine is a selective serotonin–norepinephrine reuptake inhibitor (SSNRI) indicated for treatment of depression (Goldstein, 2007), generalized anxiety disorder (GAD) (Wright & VanDenBerg, 2009) and reliefs the pain of peripheral neuropathy and fibromyalgia (Lunn et al., 2014). The drug undergoes excessive first pass metabolism resulting in poor bioavailability (≈50%) after oral administration thus making it a good candidate for administration via a buccal delivery system.

In a previous study, Peddapalli et al. (2017) prepared DH buccal patches to improve the drug bioavailability by bypassing the hepatic metabolism and acid degradation in the stomach. This study concluded that DH can be delivered via the buccal route, however, the study did not support this claim by performing pharmacokinetic studies in human subjects.

Therefore, this study is a further approach to formulate and evaluate buccal mucoadhesive films for improving the bioavailability of duloxetine. The protocol of the study includes performing a pharmacokinetic study on healthy human volunteers to compare the selected film formulation that achieves the best physicochemical characteristics with DH reference product (Cymbalta oral capsules) in order to confirm the delivery of DH via the buccal mucosa. The study also includes performing accelerated stability study to investigate the stability of DH in the selected films.

## Materials and methods

### Materials

Duloxetine hydrochloride (DH) was a kind gift from (Evapharm Pharmaceuticals, Cairo, Egypt). Hydroxypropyl methylcellulose (HPMC) 100 LV and polyvinyl alcohol (PVA) molecular weight of 22,000 Da were purchased from Sigma-Aldrich, USA. Propylene glycol (PG), disodium hydrogen phosphate, potassium dihydrogen phosphate, sodium chloride and anhydrous calcium chloride were obtained from El Nasr Pharmaceuticals, Egypt. Acetonitrile and formic acid (HPLC grade) was purchased from Merck, Germany. Cymbalta^®^ 30 mg oral capsules (Elli-Lilly, Madrid, Spain).

### Preparation of DH buccoadhesive films

Duloxetine hydrochloride buccoadhesive films were prepared adopting the solvent casting technique (Gardouh et al., 2013) using a 3^2^×2^1^mixed factorial design as shown in Table 1. HPMC was used in the concentrations (0%, 1% and 2%), while, PVA was used in the concentrations (0%, 4% and 6%). Propylene glycol was used as a plasticizer and penetration enhancer in two concentrations (10% and 30%). The specified weights of DH and PG were dissolved in distilled water in a beaker and the mixture was stirred by a magnetic stirrer until a clear homogenous solution is formed. The polymers were then added slowly (PVA first then HPMC) and stirred until a clear homogenous solution was obtained. The solutions were kept overnight to remove any entrapped air bubbles. The film solutions were casted on petri dishes (9 cm in diameter) and dried in the oven at 30 °C till complete dryness. The films were removed and 1 cm^2^ film dosage units were prepared such that each 1 cm^2^ film contained 15 mg duloxetine (equivalent to 16.85 mg DH). The films were packed in aluminum foil and stored in air tight glass containers in a desiccator at room temperature.

**Table 1.** Composition of DH buccoadhesive films.

Formula code	HPMC % (w/v)	PVA % (w/v)	PG % (w/w)	H_2_O (ml)
F1	1%	_	10%	15
F2	2%	_	10%	15
F3	_	4%	10%	15
F4	_	6%	10%	15
F5	1%	4%	10%	15
F6	2%	4%	10%	15
F7	1%	6%	10%	15
F8	2%	6%	10%	15
F9	1%	_	30%	15
F10	2%	_	30%	15
F11	_	4%	30%	15
F12	_	6%	30%	15
F13	1%	4%	30%	15
F14	2%	6%	30%	15
F15	1%	4%	30%	15
F16	2%	4%	30%	15

### Evaluation of DH buccoadhesive films

#### Visual inspection

The prepared films were visually inspected for transparency, texture and color. The ease of removal from the petri dish was also evaluated.

#### Determination of film weight and thickness uniformity

Three films (1 cm^2^ each) of every formulation were taken and weighed individually on a digital balance (Radwag Electric Balance, Mumbai, India). The average weights were calculated. The thickness of the prepared films was determined by means of micrometer (Mitutoyo, Sakado, Japan) at three different places and the mean value was calculated (Semalty et al., 2008a).

#### Determination of drug content

The drug content in the prepared films was determined by dissolving 1 cm^2^ film in simulated saliva fluid (SSF) pH 6.8 by the means of a magnetic stirrer (PMC Industries, USA) for 24 h. The solution was assayed spectrophotometerically at *λ*_max_ 290 nm against a blank of SSF pH (6.8).

#### Folding endurance

Each film was subjected to folding at the same place till it broke or folded up to 200 times (El-Nabarawi, 2005).

#### Determination of surface pH

The films to be tested were placed in a petri dish, moistened with 2 ml of distilled water and kept for 1 h. The pH was determined after bringing the electrode of the pH meter (Jenway, UK) in contact with the surface of the film and allowing equilibrating for 2 min. A mean of three readings were recorded (Bottenberg et al., 1991).

#### Determination of swelling index

The swelling studies were conducted in distilled water. 1 cm^2^ films were weighed individually (*W*_1_) and placed separately on sponge pieces in petri dishes containing 5 mL of distilled water at room temperature. At the specified times, the films were removed and excess surface water was removed carefully using filter paper then reweighed again (*W*_2_) and the swelling index (SI) was calculated using the formula (Desai & Pramod Kumar, 2004):

$$\begin{array}{l} \text{SI}\left(\%\right)=\frac{\left(W_{2}–W_{1}\right)}{W_{1}}\times 100 \end{array}$$

#### Determination of *in vitro* bioadhesion

To investigate the mucoadhesion properties of the prepared DH films, the chicken pouch membrane model was adopted as model mucosa (Wong et al., 1999; El-Samaligy et al., 2004) with a slight modification. The chicken pouches with its contents and surface fats were removed, kept frozen at –20 °C in SSF (pH 6.8), and only thawed to room temperature before the experiment was carried on. In this test, chicken pouch buccal mucosa was fixed on a glass slide using cyanoacrylate adhesive. The slide was mounted and fixed onto an inverted glass beaker which was fixed on the lower arm of the tensile strength instrument (Tinius Olsson, Redhill, England). The prepared films were fixed to another slide using cyanoacrylate adhesive. The film chicken pouch and pressed together for 2 min to facilitate adhesion. The slide on which the film was mounted was attached to the upper arm of the tensile strength instrument by a thread. The apparatus was operated such that the force required to detach the film from the chicken pouch tissue is measured and this force is a measure of the mucoadhesive strength between the film and the buccal tissue. All tests were carried out in duplicates at room temperature.

#### *In vitro* release study

The USP dissolution tester II method was used to study the drug release from the prepared films. The dissolution medium consisted of 250 ml of SSF (simulated saliva fluid) at pH 6.8 in order to obtain sink conditions. The release was performed at 37 ± 0.5 °C with a rotation speed 50 rpm. The one side of the buccal film was adhered to a 3 cm diameter glass disk using cyanoacrylate adhesive and the disk was placed at the bottom of the dissolution vessel so that the drug release is allowed only from the upper side of the film (Okamoto et al., 2001). Samples of 3 ml were withdrawn at pre-determined time intervals and replaced with fresh medium. The samples were analyzed after appropriate dilution by UV spectrophotometry (Jenway6715, Essex, UK) at *λ*_max_ 290 nm. The drug release percentage was plotted versus time. The release data were analyzed according to Peppas equation in order to determine the mechanism of drug release (Peppas, 1985).

### Statistical analysis

Test for significance for the evaluation tests was done by applying paired *t*-test (*p* < .05). The data obtained from the evaluation tests was analyzed using the statistical program Design-Expert^®^ Software, Version 7.0.

### Accelerated stability study

Samples of the selected films were stored in an oven kept at 40 °C and 75% humidity for six months. The stored films were evaluated for their physical appearance (transparency, color and texture), the increase in weight and percentage drug content. A release profile comparison was performed under identical conditions for the fresh and stored films. A simple model independent approach which uses a similarity factor (*f*_2_) to compare dissolution profiles was adopted. The similarity factor (*f*_2_) was calculated using the equation:

$$f_{2}\;=\;50\;\text{log}\left[1+1/n\;\sum nt=1\;\left(Rt\;-\;Tt\right)2\right]\;-\;0.5\;\times \;100$$

Where *Rt* is the dissolution value of the freshly prepared films at time *t*, *Tt* is the dissolution value of the stored films for six months and n is the number of time points.

### Bioavailability study of selected DH buccoadhesive film

#### Study design

The design of this study was a comparative, randomized, single dose, two-way crossover open-label study performed on two phases using the two formulations: Cymbalta^®^ 30 mg oral capsules as a reference product and the selected DH buccoadhesive film (F2) (30 mg/cm^2^ film). Six subjects were participating in this study. The volunteers were randomly numbered and divided into two dosing groups. All subjects were fasted for at least 10 h overnight the day before the study. The group receiving Cymbalta^®^ 30 mg capsule were instructed to swallow the capsule with a cup of water (240 ml), while the other group was instructed to stick the buccal film on the gingiva in the area above the canine and to press on the formula for few seconds to insure adhesion to the buccal mucosa. The volunteers were warned not to swallow the buccal films. Five milliliters venous blood samples were collected in heparinized glass tubes at zero time (pre-dose), 0.5, 1, 2, 3, 4, 5, 6, 7, 8, 9, 10, 11, 12, 24, 48 and 72 h post dose. Blood samples were collected and centrifuged at 3000 rpm for 10 min then plasma was immediately separated and frozen at −20 °C till assayed. A period of seven days was allowed between each of the two phases as a washout period. This study was conducted with the formal approval of the local human subject and ethics committee in faculty of pharmacy, Cairo University.

#### Instrumentation and chromatographic separation

The analysis was performed using a Shimadzu Prominence (Shimadzu, Japan) series LC system equipped with degasser (DGU-20A3) and solvent delivery unit (LC-20AD) with an auto-sampler (SIL-20 A). The system was used to inject 25 µl aliquots of the processed samples on a C18, 100 A (50 × 4.6 mm) (Phenomenex, USA), 5 μm particle size. A sensitive and validated LC-MS/MS method was adopted for the separation and quantitation of DH using reboxetine as an internal standard (IS) (Chandrapal Reddy et al., 2012). The employed isocratic mobile phase consisted of acetonitrile and 0.5% formic acid in water 80:20 (v/v) was pumped at 1.0 ml/min. MS/MS detection in positive ion mode using AB Sciex (Foster City, CA, USA) API-3200 mass spectrometer equipped with a Turbo Ionspray interface at 550 °C. The ion spray voltage was set at 5500 V. Detection of the ions was performed in the multiple reactions monitoring (MRM) mode, monitoring the transition of the *m/z* 298.1 precursor ion to the *m/z* 154.2 for DH and *m/z* 314.2 precursor ion to the *m/z* 175.1 for the internal standard. The analytical data were processed by Analyst software version 1.4.2 (Applied Biosystems Inc., Foster City, CA, USA).

#### Standard solution and sample preparation

To prepare the standard calibration samples, aliquots of 0.5 ml human plasma were spiked with DH stock solution (100 ng/ml) and an aliquot of 25 µL of reboxetine solution, (5 µg/ml) IS to produce calibration standards at the following concentrations: 0.1, 0.2, 1, 5, 20, 40, 60 and 70 ng/ml.

For sample preparation, 0.5 ml human plasma and 25 µl of Reboxetine solution (IS) was vortexed in 10 ml glass tubes for 1 min. Four milliters of ethylacetate were added, vortexed for another 3 min then centrifuged at 3000 rpm for 10 min. The organic layer (3 ml) was transferred to clean glass tube and evaporated to dryness using centrifugal vacuum concentrator at 45 °C. The dry residue was reconstituted in 500 µl of mobile phase and an aliquot of 20 µl of this solution was loaded into LC–MS/MS.

#### Pharmacokinetic parameters and statistical calculations

Peak concentrations (*C*_max_) and peak times (*T*_max_) were derived directly from the experimental points. The other pharmacokinetic parameters; *K*_el_, MRT, AUC_0–72_and AUC_0–∞_ were computed by noncompartmental analysis using Kinetica^®^ Software (version 4.4.1). The pharmacokinetic parameters of the two tested formulations were compared by one way ANOVA using the software SPSS (SPSS Inc., Chicago, IL, USA). The significance of the difference was determined at (*p* ≤ 0.05).

## Results and discussion

### Evaluation of DH buccoadhesive films

The visual inspection of the prepared films showed that all films were elegant in appearance, transparent, homogeneous, flexible and easily removed from the petri dishes.

The results of the physicochemical parameters of the prepared DH buccoadhesive films are presented in Table 2.

**Table 2.** The physicochemical parameters of the prepared DH buccoadhesive films.

	Thickness (mm)	Weight (mg)	% Drug content	pH	Max. Swelling index (%)	Mucoadhesion force (N)	Kinetic parameters of the release data ofDH films		
	Mean ± SD	Mean ± SD	Mean ± SD	Mean ± SD	Mean ± SD	Mean ± SD	*K*	*n*	*R* ^2^
F1	0.2 ± 0.0047	10.78 ± 0.226	98.73 ± 1.22	7.21 ± 0.034	143.84 ± 4.37	0.138 ± 0.002	0.049	0.77	0.993
F2	0.28 ± 0.0082	12.42 ± 0.286	98.51 ± 0.97	7.16 ± 0.02	218.35 ± 6.12	0.252 ± 0.003	0.030	0.901	0.973
F3	0.24 ± 0.0047	24.61 ± 0.308	98.93 ± 1.63	7.01 ± 0.07	49 ± 1.28	0.111 ± 0.001	0.219	0.424	0.991
F4	0.26 ± 0.0024	29.25 ± 0.359	99.14 ± 0.75	7.28 ± 0.05	34.83 ± 1.94	0.09 ± 0.002	0.163	0.564	0.998
F5	0.59 ± 0.0082	26.46 ± 0.393	98.82 ± 1.27	6.99 ± 0.03	27.36 ± 1.16	0.137 ± 0.003	0.092	0.508	0.978
F6	0.66 ± 0.0047	35.32 ± 0.357	99.12 ± 0.97	7.02 ± 0.06	22.51 ± 1.64	0.125 ± 0.003	0.115	0.495	0.905
F7	0.67 ± 0.0082	52.19 ± 0.531	98.98 ± 0.66	7.24 ± 0.01	30.58 ± 2.01	0.174 ± 0.003	0.058	0.519	0.995
F8	0.72 ± 0.0082	65.88 ± 0.196	99.11 ± 0.95	7.17 ± 0.02	28.76 ± 1.88	0.149 ± 0.002	0.090	0.493	0.976
F9	0.14 ± 0.0082	16.84 ± 0.481	99.16 ± 1,73	6.62 ± 0.08	135.42 ± 5.49	0.123 ± 0.001	0.077	0.749	0.998
F10	0.27 + 0.0082	19.69 ± 0.194	98.89 ± 1.96	6.86 ± 0.03	152.37 ± 6.03	0.238 ± 0.003	0.130	0.562	0.965
F11	0.21 + 0.0047	25.77 ± 0.350	98.78 ± 2.15	6.97 ± 0.03	45.28 ± 1.47	0.08 ± 0.003	0.051	0.86	0.995
F12	0.32 + 0.0216	29.95 ± 0.371	99.32 ± 1.48	7.27 ± 0.06	31.84 ± 1.21	0.075 ± 0.002	0.402	0.24	0.927
F13	0.56 + 0.0125	29.66 ± 0.460	98.78 ± 2.41	6.60 ± 0.04	25.68 ± 1.33	0.118 ± 0.003	0.044	0.832	0.955
F14	1.04 + 0.017	37.07v0.384	98.69 ± 2.33	6.21 ± 0.04	22.37 ± 1.19	0.106 ± 0.005	0.051	0.878	0.998
F15	0.77 + 0.0047	53.97 ± 0.252	99.15 ± 1.78	6.92 ± 0.03	27.58 ± 1.65	0.157 ± 0.004	0.162	0.491	0.958
F16	1.12 + 0.0094	66.94 ± 0.894	98.74 ± 2.11	6.97 ± 0.05	25.39 ± 1.78	0.133 ± 0.002	0.244	0.409	0.997

The film thicknesses were observed to be in the range of 0.2 mm (F9) to 1.12 mm (F16).

The average weight of the films was found to be in the range of 10.78 mg (F1) to 66.94 mg (F16).

Average drug content was found range between 99 ± 0.3%, hence, it is deduced that all formulations complied with the pharmacopeia specifications for drug content (85–115%) (Pharmacopoeia, 2013). Low SD in film thickness, weights and drug content results indicated uniformity of thickness and film weights and that the drug was uniformly distributed in the prepared formulations.

All prepared films resisted breakage upon folding them for more than 200 times at same place. The films did not show any cracks indicating that they possess suitable mechanical elasticity and durability over a long period of time.

### Determination of surface pH

It is desirable to keep the pH of buccal films within satisfactory limit of 7.0 ± 1.5 (Patel et al., 2006) in order to avoid any mucosal irritation and hence increase patient compliance. The pH of all prepared films lied within this range and the pH values ranged between 6.21 (F14) and 7.28 (F4) as shown in Table 2 and hence no mucosal irritation was expected from these formulations.

### Determination of swelling index

Appropriate swelling behavior of a buccoadhesive system is vital for uniform and prolonged release of drug and effective mucoadhesion (Junginger et al., 1999).

The highest swelling index was observed for F2 (218.35%), while, the lowest swelling index was observed for F14 (22.37%) as shown in Table 2.

Films prepared with HPMC showed rapid and high initial swelling with gradual increase reaching a peak within 30 min corresponding to the maximum hydration of the polymer matrix. This was followed by slight decrease in swelling index due to erosion of the films. HPMC is a hydrophilic polymer capable of effective surface wettability and, consequently, allow for water penetration within the polymer matrix, thus resulting in high swelling behavior (Soad et al., 2009). The swelling index was found to increase significantly by the increase in HPMC concentration (Koland et al., 2010a).

Films prepared with HPMC and PVA showed similar swelling behavior as the films prepared with HPMC alone, however, the value of maximum swelling was found to be less. This can be attributed to the linear chain of PVA which enables it to form hydrogen bond with HPMC chains. This interaction between the two polymers caused that the polymeric network formed become more rigid and tight and as a result the penetration of water into the polymeric network was decreased, so the swelling of these films was reduced (Saringat et al., 2005).

Films prepared with PVA alone showed low swelling index which might be due to the crystallization and the linear structure of PVA (Ochiai et al., 1974; Muppalaneni & Omidian, 2013). Crystallization makes the polymer unable to absorb large amounts of water into its structure and so prevent the polymer from efficient swelling (Muppalaneni & Omidian, 2013). The linear chain of PVA allows the formation of hydrogen bonds between the OH groups of neighboring chains and this tends to pack the polymer molecules in a compact structure even if the polymer structure is not regular (Ochiai et al., 1974). The swelling index was found to decrease significantly when the percentage of PVA in the film is increased (Singh et al., 2014; Verma & Chattopadhyay, 2014).

The increase of PG concentration led to significant decrease in the swelling index which can be attributed to the interaction of PG with the hydrophilic polymers (HPMC and PVA) via hydrogen bonding and this decreases the effective water uptake by the polymers, thus reducing the swelling of the films (Sanyang et al., 2016). Decrease in swelling due to the increase in the plasticizer concentration was reported in previous studies (Müller et al., 2008; Rajput et al., 2011).

### Determination of *in vitro* mucoadhesion

It is common that the films prepared with hydrophilic polymers possessing high swelling capability would have high mucoadhesion forces and better adherence to the oral mucosa (Gu et al., 1988). However, a critical degree of hydration exists where optimum swelling and bioadhesion occurs because if the system is overhydrated and the degree of swelling is too great, a slippy mucilage results and this can be easily detached from the buccal cavity (Peppas & Buri, 1985).

The highest mucoadhesion strength was observed with the films prepared with 2% HPMC (F2) which is in correlation with the swelling results as the F2 films showed the highest swelling among all prepared films as shown in Table 2. HPMC is a hydrophilic polymer which is capable of effective swelling and formation of effective and strong hydrogen bonding with mucin, therefore, facilitating mucoadhesion (Kim et al., 2007).

The films prepared with HPMC and PVA showed lower mucoadhesion strength than HPMC films which can be attributed to the interaction between PVA and HPMC through hydrogen bonds which decreases the available active sites on both polymers to interact with mucin which facilitates mucoadhesion, thus reducing the mucoadhesion strength of the films (Boddupalli et al., 2010).

For PVA films, the observed low mucoadhesion strength can be attributed to the low swelling of these films (Singh et al., 2014). Moreover, the mucoadhesion was found to decrease with the increase in PVA concentration (Solomonidou et al., 2001).

It was observed that increasing the concentration of PG from 10% to 30% led to decrease in mucoadhesion force of the prepared films (Rajput et al., 2011) which can be explained that PG contain hydroxyl (OH) groups that can form effective hydrogen bonding with both mucin and hydrophilic polymers such as HPMC and PVA, thus weakening the effective bonding between the polymer and mucin with the consequent decrease in mucoadhesion (Rajput et al., 2011).

### *In vitro* release study

**Figure 1.**
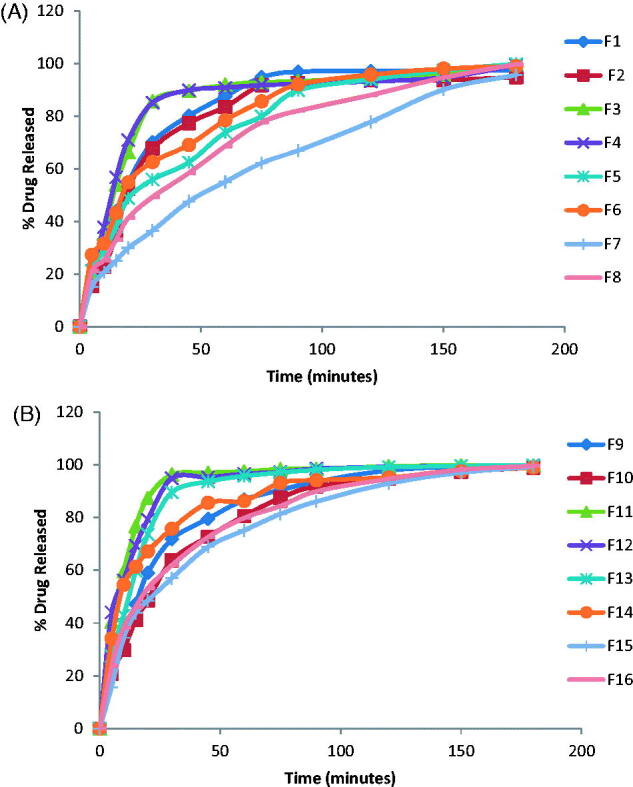
Release profiles of DH films prepared with (A) 10% PG (F1–F8) and (B) 30% PG (F9–F16).

The *in vitro* release profiles of DH from the prepared films are represented in Figure 1(A,B).

The general features of DH release profiles from the prepared films revealed that there was a high initial flash release of the drug in the first 15 min. The maximum amount of drug released was 76.65% (F12). The maximum amount of drug released after two hours was achieved by F13 (99.45%). The percentage of drug released after 2 hours exceeded 90% for all of the prepared films except F8, F7 whose drug release % was 87.91%, 77.74%, respectively. DH release from the films prepared with HPMC alone or with PVA showed gradual increase with time reaching maximum value after 90 minutes which can be attributed to the gradual swelling of HPMC in the film matrix. The release of DH was found to decrease with the increase in HPMC concentration; however, the decrease was non-significant. This decrease can be attributed to the increase in the diffusional path length and film matrix viscosity which retarded the drug release from the polymer matrix (Sekhar et al., 2008). The release results are also in accordance with the swelling results as the 2% HPMC films exhibited higher swelling index than 1% films (Vishnu et al., 2007) and maximum swelling was achieved in a gradual manner.

Films prepared with PVA alone or with HPMC showed higher drug release % than the films prepared with HPMC alone. The percentage of drug released was found to increase significantly by the increase in PVA concentration from 4% to 6%. This can be attributed to the hydrophilic and fast dissolving property of PVA with the consequent formation of pores and channels for water to diffuse into the film matrix thus increasing diffusional surface area and, hence, facilitating fast drug release (Abha et al., 2011).This is in agreement with the results obtained by Prabhu et al. (2011).

The increase of PG concentration from 10% to 30% was found to increase significantly the drug release (Bendas et al., 1995; Koland et al., 2010b). This can be explained that PG dissolves in the release medium which diffuse into the polymeric film and the water soluble plasticizer does not interfere with the movement of water molecules within the film, thus, facilitating fast wettability and dissolution of the drug and, hence its fast release from the film matrix. Moreover, PG can contribute to higher drug release by increasing the drug partition coefficient, thus increasing drug diffusion (Gardouh et al., 2013).

### Kinetic analysis of the release data

The release results were analyzed according to Peppas equation (Peppas, 1985) in order to determine the order of drug release from the polymeric blend.

$$\left(Mt/M\infty =\;Kt^{n}\right)$$

where *Mt*/*M∞* is fractional release of the drug, ‘*t*’ denotes the release time, ‘*K*’ represents a constant, incorporating structural and geometrical characteristics of the drug/polymer system and ‘*n*’ is the diffusional exponent and characterizes the type of release mechanism during the dissolution process. The values of *K*, *r*^2^ and *n* are presented in Table 2. The kinetic analysis of the DH release profiles from the prepared films showed that the values of (*n*) lied between 0.45 and 0.89 for the release of DH from all prepared films (except F2, F3, F12, and F16) indicating non-fickian release mechanism where the drug release is controlled by combination of diffusion and polymer chain relaxation mechanisms (Korsmeyer et al., 1983). For F2, *n* = 0.901 indicating super case II transport (*n* > 0.89), while, for F3 and F16, n value was less than 0.45 indicating fickian release mechanism where drug release is controlled mainly by the diffusion of the drug. For F12, the n value was 0.24 which can be attributed to the rapid dissolution and erosion of these films indicating erosion release mechanism (Semalty et al., 2008b).

### Statistical analysis

The analyzed data of the evaluation tests using the statistical program Design-Expert^®^ Software, Version 7.0 showed that the films prepared with 2% HPMC using 10% PG (F2) exhibited the best physicochemical characters for buccal administration.

### Accelerated stability study

The visual and physical inspection of the selected films (F2) stored at 40 °C temperature and 75% relative humidity for 6 months revealed no changes in the physical characteristics (transparency, texture, and color). No appreciable change in the weights and DH content which indicate high stability of the drug in the prepared formulation (Table 3).

**Table 3.** Weights and percentage drug content of the selected films (F2) stored at 40  ^°^C temperature and 75% relative humidity compared to freshly prepared films.

Time (months)		0	1	2	3	6
Weight (mg) Mean ± SD	Fresh	13.42 ± 0.29	13.33 ± 0.36	13.21 ± 0.29	13.09 ± 0.371	13.01 ± 0.42
	Stored	13.47 ± 0.33	13.36 ± 0.26	13.26 ± 0.25	13.19 ± 0.24	13.11 ± 0.19
Drug Content (%) Mean ± SD	Fresh	104.46 ± 0.34	104.26 ± 0.75	103.93 ± 1.04	103.84 ± 0.59	104.02 ± 0.69
	Stored	104.06 ± 0.14	103.57 ± 0.38	102.73 ± 0.20	102.4 ± 0.44	102.58 ± 0.41

**Figure 2.**
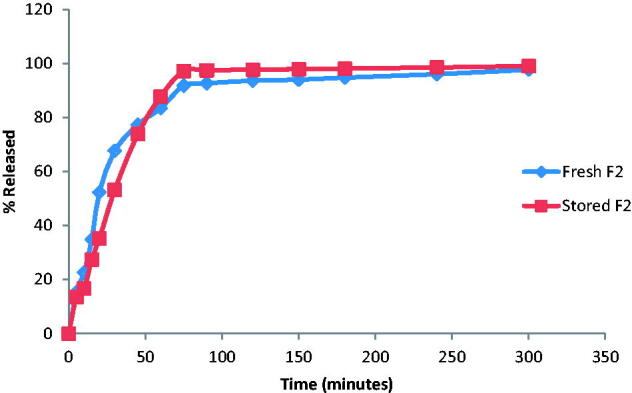
Release profile of DH from the freshly prepared and stored F2 films in SSF (pH 6.8).

Analyzing the dissolution data of stored and fresh films indicated that storing the films at the specified conditions had no marked effect on drug dissolution with similarity factor (f_2_) > 50. The release profile of DH from the fresh and stored films is represented in Figure 2.

### Bioavailability study of DH buccoadhesive films

The calibration curve of DH showed a linear response across the concentration range used from 0.1 to 70 ng/ml. Calibration curve was generated using 1/*x* weighted least squares linear regression analysis, the best fit straight line was presented by the equation: *y* = 1.012*x* − 0.319 and the determination coefficient was 0.992. The percentage recovery was found to range between 90.27% and 93.88% for the tested DH-spiked plasma (results not shown). The precision obtained had a coefficient of variance (C.V) in the range of 0.86–3.86% for inter-day precision and 0.82–7.38% for intra-day precision, respectively, which meets the FDA guidelines of not exceeding 15% for each concentration.

**Figure 3.**
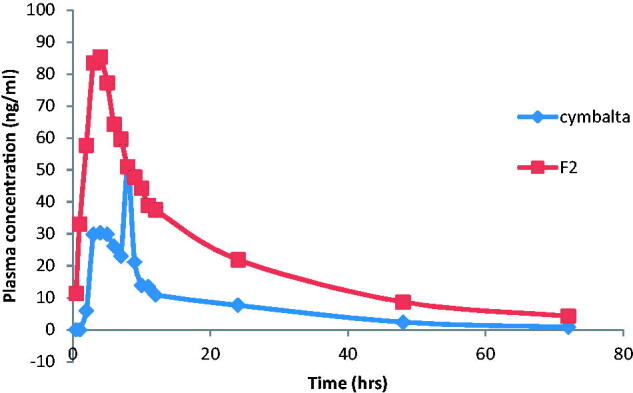
Mean plasma concentration–time profiles of DH after oral administration of Cymbalta^®^ capsules and buccal administration of F2 films to human volunteers.

The mean DH plasma concentrations following the administration of Cymbalta^®^ 30 mg oral capsules and buccal application of F2 are shown in Figure 3 and the mean pharmacokinetic parameters are reported in Table 4. Pharmacokinetic results showed that *C*_max_ of F2 was 89.33 ± 34.21 ng/ml compared to 54.1 ± 33.83 ng/ml for Cymbalta^®^. The *C*_max_ increased by ≈1.65 folds indicating improved the absorption of DH.

**Table 4.** Pharmacokinetic parameters of DH following the oral administration of Cymbalta^®^ capsules and buccal administration of F2 (Mean ± SD).

Pharmacokinetic parameter	Cymbalta^®^ 30 mg capsule	F2 films
*C*_max_ (ng/ml)	54.10 ± 33.83	89.33 ± 34.21
*T*_max_ (h)	5.67 ± 1.86	3.5 ± 0.84
AUC_0–t_ (ng h/ml)	518.5 ± 333.32	1536.8 ± 931.45
AUC_0–∞_ (ng h/ml)	535.09 ± 337.01	1646.17 ± 1043.7
MRT (h)	20.99 ± 4.8	22.36 ± 4.32
*K* (1/h)	0.05 ± 0.01	0.05 ± 0.01
Relative bioavailability (%)	–	296.39

The time required to reach maximum peak plasma concentration (*T*_max_) of DH after administration of F2 was 3.5 ± 0.34 h compared to oral Cymbalta^®^ capsules whose *T*_max_ value was 5.67 ± 0.76 h. This indicates that the buccal film F2 had more rapid onset of action and rapid absorption compared to the market product, Cymbalta^®^. The ANOVA analysis showed that there was a significant difference between the *T*_max_ of F2 and that of the market product.

The AUC_0–72_ of F2 was 1536.8 ± 931.45 ng h/ml compared to 518.5 ± 333.32 for Cymbalta 30 mg capsules, indicating nearly three folds increase. Similarly, AUC_0-∞_ for F2 was 1646.17 ± 1043.7 ng h/ml compared to 535.09 ± 337.01 ng h/ml for Cymbalta 30 mg capsules. This shows that the amount of drug absorbed via the buccal route was significantly higher than that via the oral route. The ANOVA analysis showed that there was a significant difference between the AUC_0–72_ of F2 and that of the market product. The relative bioavailability of DH from F2 was 296.39% indicating that F2 films improved the bioavailability of DH via the buccal route.

The improved rate and extent of absorption and hence the bioavailability of DH might be due to the avoidance of the first pass metabolism which is achieved through the buccal administration. Also, the prolonged *in vivo* mucoadhesion time of F2 (reported by the volunteers to exceed 3 h) facilitated continuous drug release throughout the application time. Moreover, the rapid transport of DH across the epithelial buccal mucosa into the interstitial fluid then to the venous circulation resulted in a significant shorter *T*_max_ for F2 compared to the market product (Rawas-Qalaji et al., 2006).

## Conclusions

Buccoadhesive films containing DH had been prepared with satisfactory physicochemical characters. The selected film formula (F2) was able to deliver the drug through the buccal mucosa with the substantial improvement in the relative bioavailability compared to the market product. The present study indicates a good potential of the prepared buccal buccoadhesive films containing DH for systemic delivery with added advantages of circumventing the hepatic first pass metabolism and substantial dose reduction.
